# Evaluation of Anti-Colitis Effect of KM1608 and Biodistribution of Dehydrocostus Lactone in Mice Using Bioimaging Analysis

**DOI:** 10.3390/plants9091175

**Published:** 2020-09-10

**Authors:** Sullim Lee, Sang-Back Kim, Jaemin Lee, Jimin Park, Sungyoul Choi, Gwi Seo Hwang, Han-Seok Choi, Ki Sung Kang

**Affiliations:** 1 College of Bio-Nano Technology, Gachon University, Seongnam-si, Gyeonggi-do 13120, Korea; sullimlee@gachon.ac.kr; 2 Kolmar Korea R&D Complex, Kolmar, Korea Co. Ltd, 61, Heolleung-ro 8-gil, Seocho-gu, Seoul 06500, Korea; m302@kolmar.co.kr (S.-B.K.); jimpark@kolmar.co.kr (J.P.); 3 College of Korean Medicine, Gachon University, Seongnam-si, Gyeonggi-do 13120, Korea; jaemin.lee426@gmail.com (J.L.); pc1075@gachon.ac.kr (S.C.); seoul@gachon.ac.kr (G.S.H.)

**Keywords:** inflammatory bowel disease, bioimaging, KM1608

## Abstract

Inflammatory bowel disease (IBD) is a chronic relapsing disorder modulated by numerous factors. Recent failures of drugs targeting single factors suggest that multitargeting drugs could be useful for the treatment of IBD. Natural medicines may be an alternative option for the treatment of IBD, owing to the complex nature of the disease. However, most natural medicines have poor in vitro and in vivo translational potential because of inadequate pharmacokinetic study. KM1608, a mixture of the medicinal plants *Aucklandia lappa*, *Terminalia chebula*, and *Zingiber officinale*, was examined for its anti-colitis effects and biodistribution using bioimaging. Dehydrocostus lactone, as a marker compound, was analyzed to assess the biodistribution of KM1608. KM1608 significantly attenuated the disease activity of dextran sodium sulfate-induced colitis in mice and suppressed inflammatory mediators such as myeloperoxidase, proinflammatory cytokines (TNF-α and IL-6), and the Th2-type cytokine IL-4 in the colon. Optical fluorescence imaging revealed that KM1608 was distributed in the intestinal area as a target organ. Collectively, our findings suggest that KM1608 is a potential therapeutic formulation for IBD.

## 1. Introduction

Inflammatory bowel disease (IBD), a continuous inflammatory disorder of the gut, is characterized by a dysregulated immune response and mainly encompasses Crohn’s disease and ulcerative colitis [1,2]. The prevalence of IBD has been increasing worldwide during the past few decades [3]. Although the pathogenesis of IBD has been thoroughly studied, present pharmaceutical agents still have limitations regarding therapeutic effects, dosing periods, and adverse effects [4]. There is increasing interest in alternative treatments, owing to the dissatisfaction with conventional therapeutic agents such as aminosalicylates, corticosteroids, antibodies, and immunosuppressants [5]. Additionally, recent studies on various drug targets in IBD, such as interferon-γ, interleukin (IL)-10, IL-13, IL-17, and chemokine receptor (CCR)-9, have indicated that a single-target therapeutic strategy is not effective in the treatment of IBD [6]. Therefore, a multi-target strategy, using multiple-component drugs such as natural product formulations, could be a better therapeutic option. 

The experimental animal model of IBD is an indispensable tool for the investigation of pathophysiology and development of new therapeutic formulations. However, there are difficulties with animal studies. As with humans, the diversity of animal IBD models causes variations in the severity, pathological features, immunological profile, and duration of the disease among studies [7]. Moreover, inter-animal variability, time lag from death to autopsy, subjectivity in symptom assessment, and analyzing only a small portion of sampled tissues can result in errors in data analysis. The assessment of disease activity in animal IBD models uses symptom observation and histological evaluation. Objective imaging methods, such as endoscopy, ultrasound imaging, and radiology imaging, can improve the assessment of disease activity and treatment planning and allow longitudinal monitoring in the same living subject [8]. A small animal colonoscopy is a useful option for in vivo monitoring. However, a colonoscopy in a mouse with colitis carries a high risk for mortality when the animal is under anesthesia and in a moribund condition [9]. Unlike endoscopy, non-invasive imaging systems, such as computed tomography (CT), positron emission tomography (PET), and fluorescent imaging, allow repetitive monitoring, the quantification of disease activity, and the visualization of various biological processes both in vivo and ex vivo [10,11,12].

KM1608, a mixture of *Zingiber officinale*, *Terminalia chebula*, and *Aucklandia lappa* extracts, is an herbal formulation in development for the treatment of IBD. We have previously reported two studies that describe the screening of medicinal plants for the formulation [13] and preliminary [14] in vitro and in vivo assays of KM1608. In this study, the therapeutic effect of KM1608 was evaluated in a dextran sodium sulfate (DSS)-induced colitis mouse model using 2-deoxy-2-[^18^F]fluoro-D-glucose (FDG)–PET imaging for the assessment of colitis symptoms. Additionally, we determined more mediators involved in IBD such as IL-17, IL-10, and IL-4—as well as MPO, TNF-α, and IL-6, which were tested in a preceding study. In this study, the determination of inflammatory mediators was performed. Furthermore, we analyzed the in vivo and ex vivo distribution of dehydrocostus lactone, a marker compound for KM1608, using optical fluorescence imaging.

## 2. Results and Discussion

### 2.1. Therapeutic Effects of KM1608 on DSS-Induced Colitis in Mice

**Figure 1:**
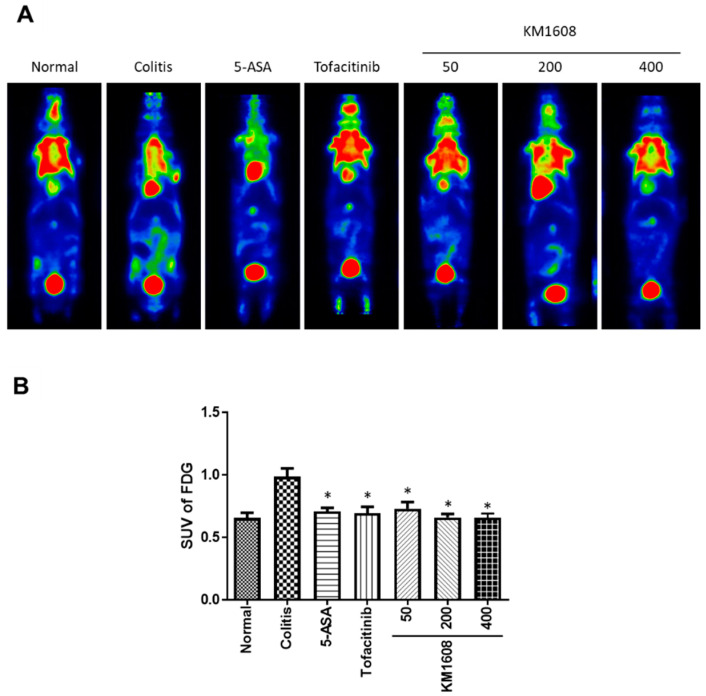
2-deoxy-2-[18F]fluoro-D-glucose (FDG)–positron emission tomography (PET) imaging in dextran sodium sulfate (DSS)-induced colitis. Colitis was induced in C57BL/6 mice by the addition of 1.5% DSS in drinking water for 7 days. Animals were treated with KM1608 (50, 200, and 400 mg/kg), 5-ASA (200 mg/kg), and tofacitinib (10 mg/kg) orally once a day. 5-ASA and tofacitinib were used as reference drugs. PET images were obtained on Day 7 before euthanasia (**A**). FDG uptake in the lower gut area was higher in colitis mice than in normal mice, and the 5-ASA-, tofacitinib-, and KM1608-treated mice showed a lower FDG uptake in the lower gut than the other colitis mice. FDG uptake in the lower gut area was quantified (**B**). Data are presented as the mean ± SEM. *n* = 8. * *p* < 0.05 vs. colitis.

To evaluate the therapeutic effect of KM1608 in IBD, we used a 1.5% DSS-induced colitis C57BL/6 mouse model. KM1608 (50, 200, and 400 mg/kg), 5-ASA (200 mg/kg), and tofacitinib (10 mg/kg) were orally administered once per day during the 7 days of 1.5% DSS/drinking water administration. 5-ASA, an aminosalicylate, and tofacitinib, a Janus kinase inhibitor, were used as reference drugs. After colitis symptoms were assessed on Day 7, all the mice were injected with FDG (0.2 mCi/kg) via the tail vein under anesthesia with 1.5% isoflurane, and FDG-PET images were taken at 1 h after FDG injection. FDG, a glucose analog, is used for the assessment of glucose metabolism in the brain, heart, and lungs. As inflammatory and neoplastic cells use glucose as an energy source in metabolic bursts, accumulated FDG is observed in the lesion sites of inflammatory diseases and cancers [15]. Thus, as a non-invasive imaging method, FDG-PET can be used to measure disease activity in human IBD and experimental IBD models [16,17]. The PET signal in the abdominal area was higher in colitis mice than in normal mice. Thus, a high uptake of FDG occurred in the inflamed intestine induced by DSS. KM1608- and reference drug-treated mice showed lower PET signals in the abdominal area than colitis mice (Figure 1A). After the selection of a region of interest (ROI) around the abdominal area in the images, the standard uptake value (SUV) for FDG was calculated to quantify the PET signal. KM1608-treated mice had significantly lower FDG uptake than colitis mice in a dose-dependent manner (Figure 1B). 

**Figure 2:**
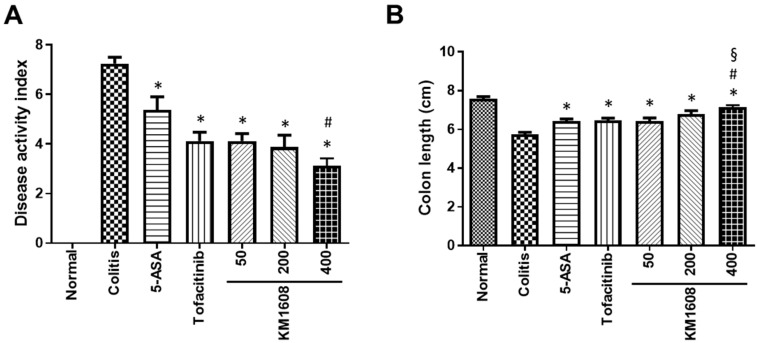
KM1608 improves the symptoms of DSS-induced colitis. Animals were treated with KM1608 (50, 200, and 400 mg/kg), 5-ASA (200 mg/kg), and tofacitinib (10 mg/kg) orally once a day. 5-ASA and tofacitinib were used as reference drugs. The disease activity index (DAI) was scored on Day 7 (**A**). Colon length was measured at autopsy on Day 7 (**B**). Data are presented as the mean ± SEM. *n* = 8. * *p* < 0.05 vs. colitis. # *p* < 0.05 vs. 5-ASA. § *p* < 0.05 vs. tofacitinib.

**Figure 3:**
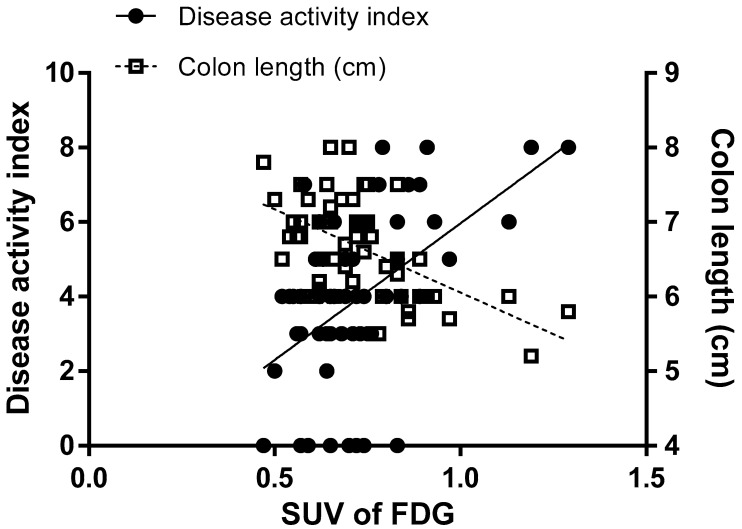
Scatter plot of the disease activity index (DAI) and colon length against the standard uptake value (SUV) for FDG. The Pearson correlation coefficient between the SUV for FDG and DAI was r = 0.5248 and *p* < 0.0001, and that between the SUV for the FDG and colon length was r = −0.5548 and *p* < 0.0001.

The assessment of colitis symptoms was performed according to the disease activity index (DAI) and length of the colon. The colitis group showed higher DAIs (Figure 2A) and shorter colons than the normal mice (Figure 2B). KM1608 administration resulted in significantly lower DAIs (Figure 2A) and significantly longer colons than those in the colitis mice (Figure 2B) in dose-dependent manners. The administration of each reference drug also significantly improved both the DAI and colon length compared with those in the colitis group. After these data were obtained, we intra-individually correlated FDG uptake with the DAI and colon length. The DAI was significantly correlated with FDG uptake (r = 0.5248 and *p* < 0.0001; Figure 3), and colon length was also significantly correlated with FDG uptake (r = −0.5548 and *p* < 0.0001; Figure 3). These correlations indicate that FDG-PET imaging can be used as an assessment method for IBD in live animals and may be useful for the longitudinal monitoring of long-term colitis models.

**Figure 4:**
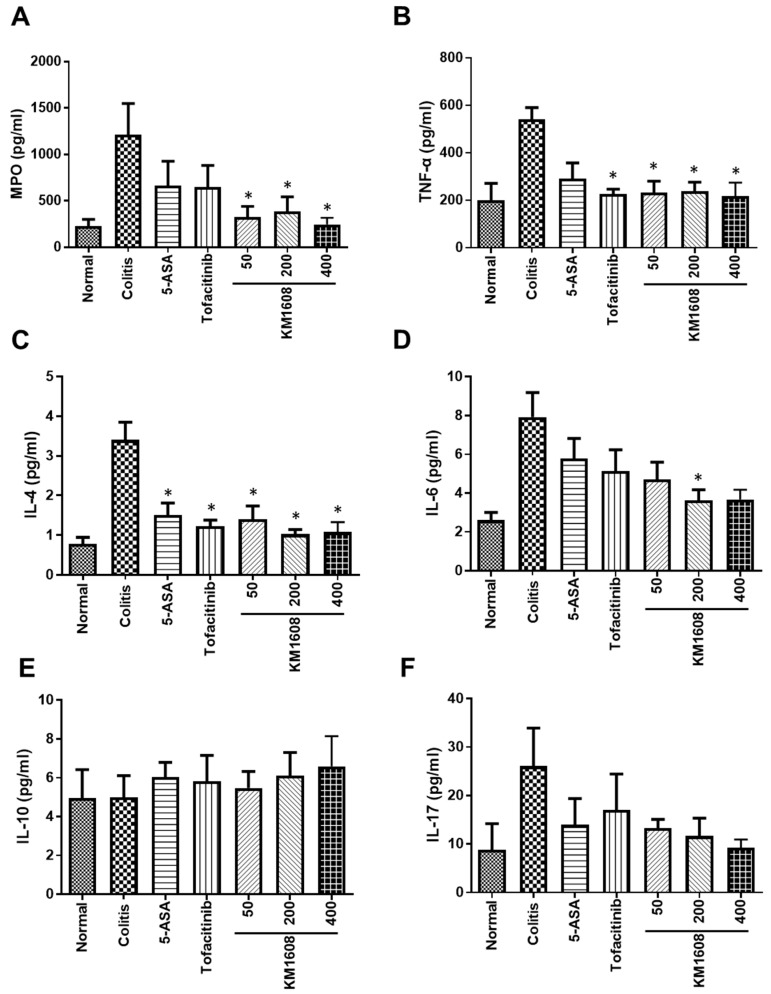
Myeloperoxidase (MPO) and cytokine (TNF-α, IL-4, IL-6, IL-10, and IL-17) levels in the colitis-induced colon were measured by ELISA. MPO (**A**), TNF-α (**B**), IL-4 (**C**), IL-6 (**D**), and IL-17 (**F**) levels were lower in KM1608 treatment than those in colitis mice. IL-10 was not changed in the colon (**E**). Data are presented as the mean ± SEM. *n* = 3–5, * *p* < 0.05 vs. colitis.

We measured MPO, TNF-α, IL-4, IL-6, IL-10, and IL-17 levels in the colon sample using commercial ELISA kits (Figure 4). MPO is a peroxidase abundantly expressed in neutrophils, which rapidly infiltrates the inflamed site [18]. Thus, MPO was measured as a marker of inflammation. Colon tissue MPO, TNF-α, IL-4, IL-6, and IL-17 levels were higher in the colitis group than in the normal group (Figure 4A–D,F), whereas the level of IL-10 was not different between these groups (Figure 4E).

IL-10 is a regulatory cytokine that suppresses proinflammatory cytokine production to maintain homeostasis [19]. A previous study has reported that C57BL/6 IL-10-deficient mice show more exacerbated colitis upon DSS administration than wild-type mice [20]. However, another study has shown that the colonic IL-10 level is elevated with the development of DSS-induced colitis in BALB/c mice [21]. At first, we speculated that the level of IL-10 would be lower in colitis mice than in normal mice, owing to homeostatic imbalance; however, there was no difference in the IL-10 level among all the groups. These discrepancies between studies may be caused by differences in animal strain, sex, and DSS concentration. The oral administration of KM1608 (50, 200, and 400 mg/kg) resulted in significantly lower MPO, TNF-α, and IL-4 levels in the colon than those in the colitis mice (Figure 4A–C). The colonic IL-6 level was lower in the KM1608-treated groups (50 and 400 mg/kg) than in the colitis mice but only significantly so with 200 mg/kg KM1608 (Figure 4D). Moreover, the IL-17 level was lower in the KM1608-treated groups (50, 200, and 400 mg/kg) than in the colitis group, but there was no statistical significance (Figure 4F). Collectively, our results demonstrate that KM1608 ameliorates DSS-induced colitis via the significant suppression of MPO, proinflammatory cytokines (TNF-α and IL-6), and T helper 2-type cytokine (IL-4) levels in the inflamed colon. These conclusions indicate that KM1608 possesses potential therapeutic effects against IBD. 

### 2.2. Biodistribution Analysis Using Optical Fluorescence Imaging

**Figure 5:**
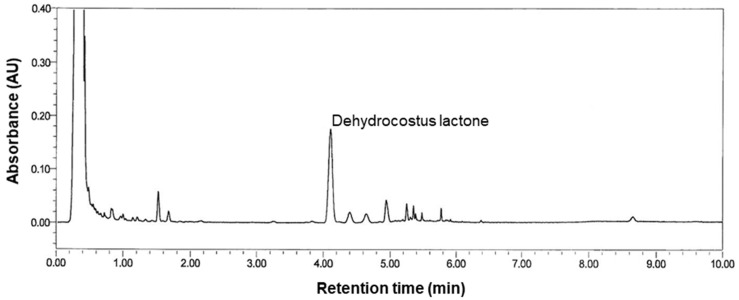
HPLC chromatogram of KM1608. The peak of dehydrocostus lactone in KM1608 was identified by comparison with the reference compound.

KM1608 is a mixture of three medicinal plant extracts, *Zingiber officinale*, *Terminalia chebula*, and *Aucklandia lappa* [13]. Natural medicines, such as KM1608, are complex mixtures of diverse compounds. Contrary to the situation with chemical drugs, it is difficult to elucidate the specific drug targets or pharmacokinetic profiles of natural medicines [22,23]. Thus, at least one putative active compound must be chosen to conduct a pharmacokinetic study [24,25]. In this study, we chose dehydrocostus lactone as a marker compound to analyze the biodistribution of KM1608 to understand whether the gastrointestinal tract was the target organ of KM1608. The representative HPLC chromatogram is shown in Figure 5. Dehydrocostus lactone is an active compound from *Aucklandia lappa* and inhibits lipopolysaccharide (LPS)-induced TNF-α production in RAW 264.7 cells and mice [26]. Moreover, it has recently been reported that the oral administration of dehydrocostus lactone ameliorates DSS-induced colitis via the reduction of the expression of proinflammatory cytokines, such as TNF-α, IL-1β, IL-6, and IL-17 [27,28]. 

**Figure 6:**
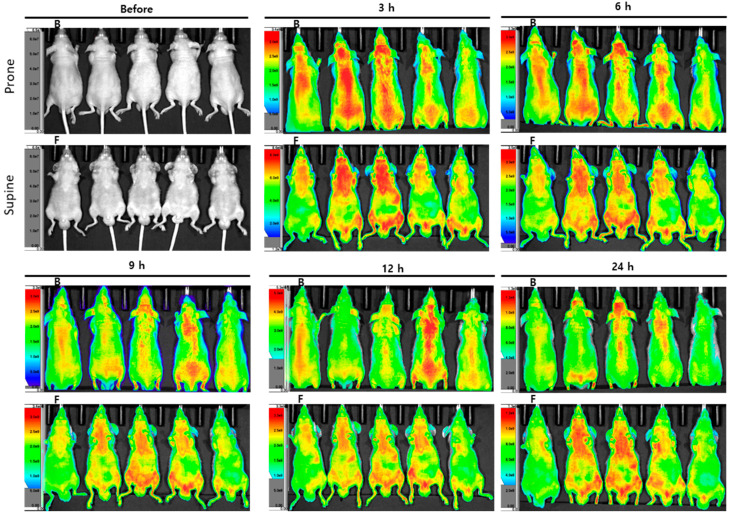
In vivo images of dehydrocostus lactone biodistribution. Image data of dehydrocostus lactone fluorescence at each time point after injection of Flamma^®^ 675 thiol-conjugated dehydrocostus lactone. The fluorescence intensity was too strong in the skin area to distinguish the distribution in internal organs.

Dehydrocostus lactone was labeled with Flamma^®^ 675 thiol and injected into mice for detection by optical fluorescence imaging. The in vivo images were scanned 3, 6, 9, 12, and 24 h after the dehydrocostus lactone injection. The fluorescence intensity was extremely strong in the skin area, even after 24 h (Figure 6, *n* = 5). Thus, it was impossible to determine where KM1608 distributed. The concentration of dehydrocostus lactone injected might have been too high to distinctively detect fluorescence in the in vivo observation. Consequently, we collected organs (hearts, lungs, livers, gastrointestinal tracts, spleens, and kidneys) from the mice and conducted ex vivo imaging from 24 h after injection.

**Figure 7:**
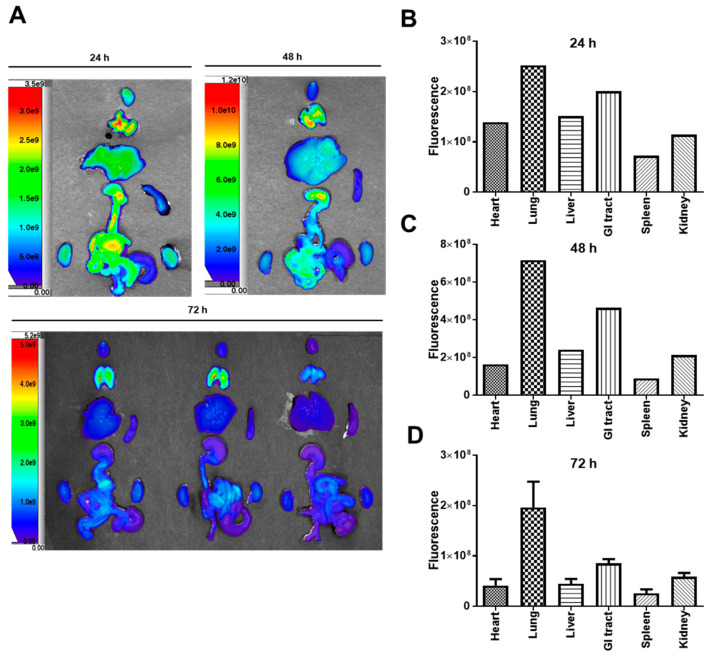
Ex vivo images of dehydrocostus lactone biodistribution. Flamma^®^ 675 thiol-conjugated dehydrocostus lactone fluorescence images of organs at each time point (**A**). Fluorescence intensity was quantified 24 (**B**), 48 (**C**), and 72 h (**D**) after dehydrocostus lactone injection.

Ex vivo images were obtained 24, 48, and 72 h after the initial dehydrocostus lactone injection (Figure 7A), and the fluorescence intensity was quantified. One mouse each was used at the 24 and 48 h time points (a total of two mice), and three mice were used at the 72 h time point. The quantified fluorescence intensity was higher in the lung and gastrointestinal tract than in other organs (Figure 7B–D). The site in which most dehydrocostus lactone accumulated was the lung. A recent study revealed that dehydrocostus lactone was effective in the attenuation of LPS-induced acute lung injury in mice via the inhibition of nuclear factor-κB [29,30]. Thus, our present results may support those of this previous study. Although the fluorescence intensity was not the highest in the gastrointestinal tract, our present study illustrated that this organ is one of the target organs of KM1608. We have plans to conduct a future study with other marker compounds and methods to understand the detailed pharmacokinetic and biodistribution profiles of KM1608.

## 3. Materials and Methods 

### 3.1. Preparation of KM1608

The method of preparing KM1608 has been described for our previous study [13,14]. Briefly, all the medicinal plants comprising KM1608 were purchased from Songrim Muyak (Seoul, Korea). *Zingiber officinale, Terminalia chebula*, and *Aucklandia lappa* were mixed in a 1:2:2 ratio before the extraction process. The mixture was extracted twice with 50% ethanol (*v*/*v*) at 80 °C for 3 h, and then, the extracted solution was filtered and evaporated for freeze drying. After the freeze drying, the obtained powder was used as the KM1608. The yield of KM1608 was 35–45%. Additionally, three medicinal plant specimens that met the standards set by Korean Pharmacopoeia were selected for the preparation of KM1608. Dehydrocostus lactone of *Aucklandia lappa*, 6-gingerol of *Zingiber officinale*, and ellagic acid of *Terminalia chebula* were used as marker compounds for the standardization of KM1608. The extracts that showed correlation coefficients of profiles r > 0.9 over the preceding 3 years were defined as KM1608.

### 3.2. Animals

All the animals were housed under standard conditions of temperature (23 ± 2 °C) and humidity (50 ± 5%) with a light/dark cycle of 12/12 h and allowed free access to food and water. All animal studies were performed in accordance with the instructions of the Institutional Animal Care and Use Committee (IACUC) of KBIO Health (Cheongju, Korea) for the colitis study (approved number: KBIO-IACUC-2018-106) and Konkuk University (Seoul, Korea) for the biodistribution study (approved number: KU19031).

### 3.3. DSS-Induced Colitis in Mice

Colitis was induced in 7-week-old female C57BL/6 mice (Koatech, Pyeongtaek, Korea) by the addition of 1.5% (*w*/*v*) DSS (160110, MP Biomedicals, Santa Ana, CA, USA) to drinking water for 7 days. In total, 56 animals were divided into 7 groups (normal, colitis, 5-aminosalicylic acid (5-ASA), tofacitinib, and three doses of KM1608, *n* = 8). The normal group received drinking water without DSS, and the colitis-induced groups (colitis, 5-ASA, tofacitinib, and KM1608) received 1.5% DSS in drinking water. 5-ASA (200 mg/kg), tofacitinib (10 mg/kg), and KM1608 (50, 200, and 400 mg/kg) were orally administered once daily to colitis-induced mice for 7 days. 5-ASA and tofacitinib were used as reference drugs. For administration, 0.5% carboxymethylcellulose solution was used as a vehicle. The employed method for assessing the symptoms of colitis was derived from published literature and used with minor modifications [31]. On Day 7, the symptoms of colitis were assessed using the disease activity index (DAI) according to the following criteria: stool consistency—0, normal; 2, mild diarrhea; 4, severe diarrhea—and rectal bleeding: 0, normal; 2, mild bleeding; 4, severe bleeding. After CO_2_-induced euthanasia, the colon was collected to measure colon length and various inflammatory mediators. 

### 3.4. FDG-PET Imaging in Colitis-Induced Mice

PET scanning was performed using a NanoScan^®^ PET/CT (Mediso Ltd., Budapest, Hungary). On Day 7, mice were anesthetized with 1.5% isoflurane in oxygen/nitrous oxide (30:70) in the imaging chamber of the NanoScan^®^ PET/CT, with body temperature and breathing rate monitoring. The PET image was acquired for 20 min at 1 h after the intravenous injection of FDG (0.2 mCi/kg). The image was reconstructed using the Monte Carlo-based 3D iterative algorithm Tera-Tomo™ engine. FDG uptake in the intestine was measured by drawing a region of interest (ROI) around the abdominal area. The standard uptake value (SUV) for FDG in the ROI was calculated with the following equation: SUV = FDG tissue concentration/(FDG dose/bodyweight).

### 3.5. Assessment of Inflammatory Mediators in the Colon of Colitis Mouse

The collected colon samples were homogenized using a homogenizer (Scilogex, Rocky Hill, CT, USA) with a lysis buffer (Intron, Seoul, Korea). The homogenized sample was centrifuged at 10,000 rpm at 4 °C for 20 min, and the supernatant was obtained. Myeloperoxidase (MPO; DY3667, R&D Systems, Minneapolis, MN, USA), tumor necrosis factor-α (TNF-α) (DY417, R&D Systems, Minneapolis, MN, USA), interleukin (IL)-4 (DY404, R&D Systems, Minneapolis, MN, USA), IL-6 (DY406, R&D Systems, Minneapolis, MN, USA), IL-10 (DY410, R&D Systems, Minneapolis, MN, USA), and IL-17 (DY421, R&D Systems, Minneapolis, MN, USA) were measured using commercial enzyme-linked immunosorbent assay (ELISA) kits according to the manufacturer’s instructions. 

### 3.6. High-Performance Liquid Chromatography (HPLC) Analysis

The HPLC profile was obtained using the Waters UPLC system (Milford, MA, USA) and Waters Acquity UPLC HSS T3 Column (2.1 × 100 mm, 1.8 μm; Milford, MA, USA) at 40 °C. Acetonitrile (A) and water containing 0.1% phosphoric acid (B) were used as the mobile phase with gradient elution (45% A at 0–4 min, 50% A at 4–5 min, and 100% A at 5–10 min). The flow rate was 0.7 mL/min, and the injection volume was 2 μL. Ultraviolet detection was performed at 220 nm to detect dehydrocostus lactone. 

### 3.7. Dehydrocostus Lactone Biodistribution

Seven-week-old male BALB/c nude mice (Orient Bio, Sungnam, Korea) were used for the evaluation of the biodistribution of dehydrocostus lactone. Dehydrocostus lactone was conjugated with Flamma^®^ 675 thiol (prepared by BioActs, Incheon, Korea) for image scanning. The conjugate was purified and analyzed by TLC, HPLC, and LC-MS by BioActs. The image was obtained by using the IVIS^®^ Spectrum in vivo imaging system (PerkinElmer, Waltham, MA, USA) and Living Image^®^ software (PerkinElmer, Waltham, MA, USA). Flamma^®^ 675 thiol-probed dehydrocostus lactone was injected into the tail vein (8 mg/mL, 100 μL). In vivo image scanning was performed 3, 6, 9, 12, 24, 48, and 72 h, and ex vivo image scanning of the heart, lung, liver, gastrointestinal tract, spleen, and kidney was performed 24, 48, and 72 h after injection. The fluorescence of the image was normalized by the intensity/ROI (region of interest) of each organ, and it was calculated automatically by the software.

### 3.8. Statistical Analysis 

Data are expressed as the mean ± SEM. Statistical analysis was performed using one-way analysis of variance (ANOVA) followed by Tukey’s post-hoc test. For correlations between the SUV for FDG and DAI, and the SUV for FDG and colon length, the Pearson correlation coefficient (r) and two-tailed test were used. Statistical significance was defined as *p* < 0.05.

## 4. Conclusions

In this study, KM1608 attenuated disease activities, observed by both conventional and bioimaging methods. This amelioration was associated with the suppression of inflammatory mediators, such as MPO, TNF-α, and IL-6. Furthermore, the fluorescence imaging results suggested that the gastrointestinal tract is a target organ of KM1608. Although the underlying mechanisms and pharmacokinetic properties must be elucidated in more detail, our results suggest that KM1608 may serve as a promising therapeutic formulation for the treatment of IBD.

## References

[1] Baumgart D.C., Carding S.R. (2007). Inflammatory bowel disease: Cause and immunobiology. Lancet.

[2] De Souza H.S.P., Fiocchi C. (2016). Immunopathogenesis of IBD: Current state of the art. Nat. Rev. Gastroenterol. Hepatol..

[3] Li F.X., Verhoef M.J., Best A., Otley A., Hilsden R.J. (2005). Why patients with inflammatory bowel disease use or do not use complementary and alternative medicine: A Canadian national survey. Can. J. Gastroenterol..

[4] Ponder A., Long M.D. (2013). A clinical review of recent findings in the epidemiology of inflammatory bowel disease. Clin. Epidemiol..

[5] Mowat C., Cole A., Windsor A., Ahmad T., Arnott I., Driscoll R., Mitton S., Orchard T., Rutter M., Younge L. (2011). Guidelines for the management of inflammatory bowel disease in adults. Gut.

[6] Bilsborough J., Targan S.R., Snapper S.B. (2016). Therapeutic targets in inflammatory bowel disease: Current and future. Am. J. Gastroenterol. Suppl..

[7] Valatas V., Vakas M., Kolios G. (2013). The value of experimental models of colitis in predicting efficacy of biological therapies for inflammatory bowel diseases. Am. J. Physiol. Gastrointest. Liver Physiol..

[8] Baumgart D.C., Sandborn W.J. (2007). Inflammatory bowel disease: Clinical aspects and established and evolving therapies. Lancet.

[9] Becker C., Fantini M.C., Neurath M.F. (2006). High resolution colonoscopy in live mice. Nat. Protoc..

[10] Freise A.C., Zettlitz K.A., Salazar F.B., Tavaré R., Tsai W.-T.K., Chatziioannou A.F., Rozengurt N., Braun J., Wu A.M. (2018). Immuno-PET in inflammatory bowel disease: Imaging CD4-positive T cells in a murine model of colitis. J. Nucl. Med..

[11] Bettenworth D., Reuter S., Hermann S., Weckesser M., Kerstiens L., Stratis A., Nowacki T.M., Ross M., Lenze F., Edemir B. (2013). Translational 18F-FDG PET/CT imaging to monitor lesion activity in intestinal inflammation. J. Nucl. Med..

[12] Lai C.P., Mardini O., Ericsson M., Prabhakar S., Maguire C., Chen J.W., Tannous B.A., Breakefield X.O. (2014). Dynamic biodistribution of extracellular vesicles in vivo using a multimodal imaging reporter. ACS Nano.

[13] Lee J., Choi H.-S., Lee J., Park J., Kim S.-B., Shin M.-S., Lee S., Hwang G.S., Koo B.A., Kang K.S. (2019). Preparation of herbal formulation for inflammatory bowel disease based on in vitro screening and in vivo evaluation in a mouse model of experimental colitis. Molecules.

[14] Shin M.-S., Kim S.-B., Lee J., Choi H.-S., Park J., Park J., Park J., Lee S., Hwang G., Koo B. (2018). Beneficial effect of herbal formulation KM1608 on inflammatory bowl diseases: A preliminary experimental study. Molecules.

[15] Glaudemans A.W.J.M., de Vries E.F.J., Galli F., Dierckx R.A.J.O., Slart R.H.J.A., Signore A. (2013). The use of (18)F-FDG-PET/CT for diagnosis and treatment monitoring of inflammatory and infectious diseases. Clin. Dev. Immunol..

[16] Treglia G., Quartuccio N., Sadeghi R., Farchione A., Caldarella C., Bertagna F., Fania P., Cistaro A. (2013). Diagnostic performance of Fluorine-18-Fluorodeoxyglucose positron emission tomography in patients with chronic inflammatory bowel disease: A systematic review and a meta-analysis. J. Crohn’s Colitis.

[17] Brewer S., McPherson M., Fujiwara D., Turovskaya O., Ziring D., Chen L., Takedatsu H., Targan S.R., Wei B., Braun J. (2008). Molecular imaging of murine intestinal inflammation with 2-deoxy-2-[18F]fluoro-D-glucose and positron emission tomography. Gastroenterology.

[18] Wéra O., Lancellotti P., Oury C. (2016). The dual role of neutrophils in inflammatory bowel diseases. J. Clin. Med..

[19] Neurath M.F. (2014). Cytokines in inflammatory bowel disease. Nat. Rev. Immunol..

[20] Li B., Alli R., Vogel P., Geiger T.L. (2014). IL-10 modulates DSS-induced colitis through a macrophage-ROS-NO axis. Mucosal Immunol..

[21] Tomoyose M., Mitsuyama K., Ishida H., Toyonaga A., Tanikawa K. (1998). Role of interleukin-10 in a murine model of dextran sulfate sodium-induced colitis. Scand. J. Gastroenterol..

[22] Hao H., Zheng X., Wang G. (2014). Insights into drug discovery from natural medicines using reverse pharmacokinetics. Trends Pharmacol. Sci..

[23] Choi J.H., Jang M., Nah S.Y., Oh S., Cho I.H. (2018). Multitarget effects of Korean Red Ginseng in animal model of Parkinson’s disease: Antiapoptosis, antioxidant, antiinflammation and maintenance of blood-brain barrier integrity. J. Ginseng Res..

[24] Bhattaram V.A., Graefe U., Kohlert C., Veit M., Derendorf H. (2002). Pharmacokinetics and bioavailability of herbal medicinal products. Phytomedicine.

[25] Trinh T.A., Park E.-J., Lee D., Song J.H., Lee H.L., Kim K.H., Kim Y., Jung K., Kang K.S., Yoo J.-E. (2019). Estrogenic activity of Sanguiin H-6 through activation of estrogen receptor α Coactivator-binding Site. Nat. Prod. Sci..

[26] Lee H.J., Kim N.Y., Jang M.K., Son H.J., Kim K.M., Sohn D.H., Lee S.H., Ryu J.H. (1999). A sesquiterpene, dehydrocostus lactone, inhibits the expression of inducible nitric oxide synthase and TNF-alpha in LPS-activated macrophages. Planta Med..

[27] Lee D., Lee D.S., Jung K., Hwang G.S., Lee H.L., Yamabe N., Lee H.J., Eom D.W., Kim K.H., Kang K.S. (2018). Protective effect of ginsenoside Rb1 against tacrolimus-induced apoptosis in renal proximal tubular LLC-PK1 cells. J. Ginseng Res..

[28] Zhou Q., Zhang W.-X., He Z.-Q., Wu B.-S., Shen Z.-F., Shang H.-T., Chen T., Wang Q., Chen Y.-G., Han S.-T. (2020). The possible anti-inflammatory effect of dehydrocostus lactone on DSS-induced colitis in Mice. Evid.-Based Complementary Altern. Med..

[29] Roy A., Park H.-J., Jung H.A., Choi J.S. (2018). Estragole exhibits anti-inflammatory activity with the regulation of NF-κB and Nrf-2 signaling pathways in LPS-induced RAW 264.7 cells. Nat. Prod. Sci..

[30] Nie Y., Wang Z., Chai G., Xiong Y., Li B., Zhang H., Xin R., Qian X., Tang Z., Wu J. (2019). Dehydrocostus lactone suppresses LPS-induced acute lung injury and macrophage activation through NF-κB signaling pathway mediated by p38 MAPK and Akt. Molecules.

[31] Wirtz S., Neufert C., Weigmann B., Neurath M.F. (2007). Chemically induced mouse models of intestinal inflammation. Nat. Protoc..

